# Isolated external jugular thrombophlebitis secondary to acute pharyngitis: a case report and a review of the literature

**DOI:** 10.1186/s13052-024-01760-4

**Published:** 2024-09-16

**Authors:** Uche C. Ezeh, Naomi Tesema, Sukaina Hasnie, Philip J. Kahn, Max M. April

**Affiliations:** 1 Division of Pediatric Otolaryngology, Department of Otolaryngology Head and Neck Surgery, NYU Langone Health, 240 East 38th Street 14th floor, New York, NY 10016 USA; 2 Department of Pediatrics, Hassenfeld Children’s Hospital at NYU Langone Health, New York, NY USA

**Keywords:** Thrombophlebitis, Streptococcus infection, Jugular vein thrombosis

## Abstract

**Background:**

External Jugular Thrombophlebitis (EJT) is a rare clinical phenomenon with few reports in the literature, especially in the pediatric population. This is a report of an unusual case of right-sided EJT in a pediatric patient secondary to acute pharyngitis with sinusitis most prominent on the left side.

**Case presentation:**

A 13-year-old presented to the emergency department with worsening upper respiratory infectious (URI) symptoms and facial swelling, cough, throat pain, and emesis. The patient had traveled to Switzerland and received amoxicillin for strep throat 6 weeks before this hospitalization. Physical examination revealed nasal purulence, allodynia over the right side of the face without overlying erythema, and oropharyngeal exudate. CT scan revealed left-sided predominate sinusitis and right external jugular vein thrombosis. Blood cultures confirmed the presence of group A streptococcus infection. Treatment included IV antibiotics, non-steroidal anti-inflammatory drugs (NSAIDs), IV steroids, and anticoagulation. Follow-up imaging demonstrated improvement in thrombosis, cellulitis, and sinus disease. The patient was discharged on antibiotics for 6 weeks and anticoagulation for 10 weeks. Follow-up imaging at 6 months revealed no EJT, and medications were discontinued.

**Conclusions:**

EJT is a rare condition, and to our knowledge, no reports of EJT with sinusitis most pronounced on the contralateral side have been published. Physicians will benefit from noting clinical signs of EJT such as facial edema, headache, erythema, and palpable neck mass, especially if these symptoms occur with URI symptoms refractory to treatment. The use of anticoagulation is controversial for internal jugular vein thrombosis, and while no guidelines for EJT exist, anticoagulation is likely not necessary save for severe complications.

## Background

Jugular venous thrombosis is a severe condition primarily affecting the internal jugular vein (IJV), which originates at the jugular foramen and forms part of the carotid sheath. The most common causes of IJV thrombosis are cancer and central venous catheter use, with trauma, infection, and IV drug abuse also identified as contributing factors [1]. 

In contrast, external jugular vein (EJV) thrombosis is a rare clinical phenomenon, and there is a scarcity of published studies on this topic [2]. The leading causes of EJV thrombosis in adults include trauma, malignancy, catheterization, head and neck infections, intravenous drug use, and compression at the affected site. Additionally, factors such as age, gender, and obesity may potentially play a role [2]. Due to the infrequency of this condition, there is a lack of consensus regarding the optimal management strategies, and no controlled studies have been conducted in pediatric populations. In this report, we present the case of a pediatric patient who presented with isolated EJV thrombophlebitis (inflammation of a vein related to blood clot formation) secondary to acute pharyngitis with intracranial complications. We also provide a comprehensive review of the existing literature on cases of isolated EJV thrombophlebitis.

## Case presentation

**Fig. 1:**
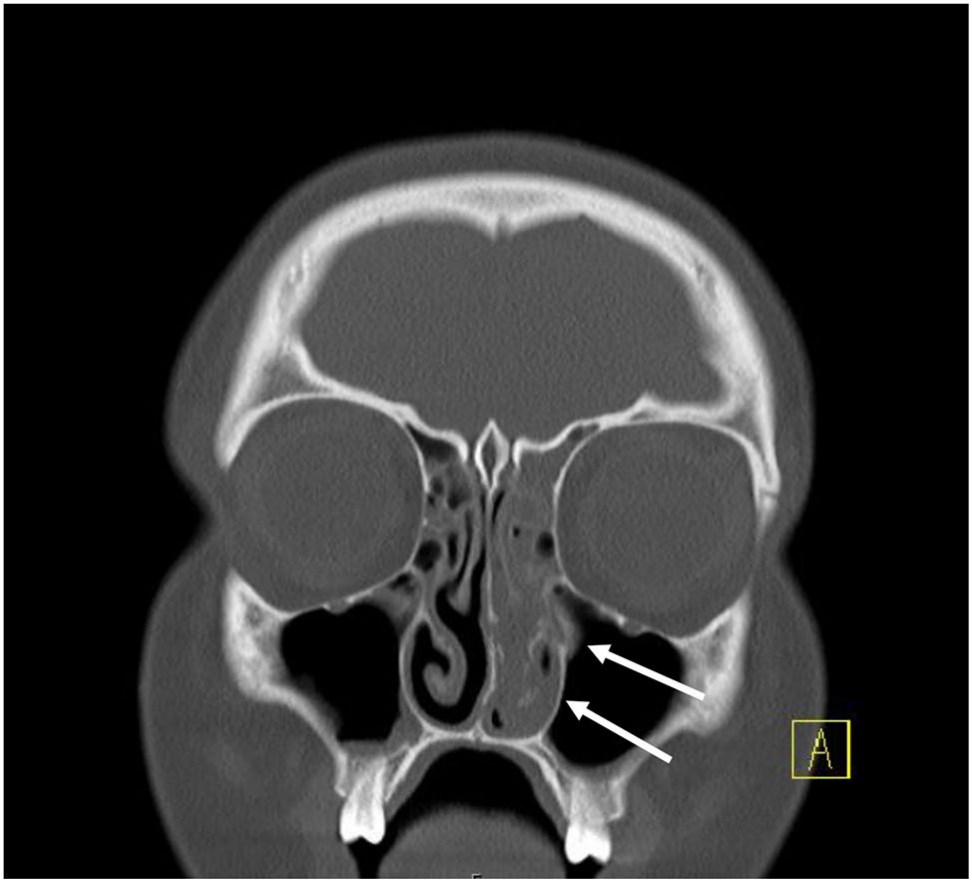
CT scan without contrast illustrating coronal image with sinusitis more severe on the left side (white arrows)

A 13-year female patient with mild oligoarticular juvenile idiopathic arthritis (JIA), with no history of immunosuppressive medication, presented to the emergency department (ED) with right facial swelling, cough, throat pain, bilateral frontal headache, and non-bloody emesis. She had traveled to Switzerland for 6 weeks before the hospitalization and tested positive for strep throat, for which she received 10 days of Augmentin treatment. Although her sore throat initially appeared to improve, it subsequently recurred along with pain and swelling over the right side of the face three days before she visited the ED. At an outpatient clinic, she exhibited a high fever (104 °F), and the physical examination revealed pain with neck motion in all directions, trismus, and erythema of the posterior oropharynx without exudates or petechiae. A strep test confirmed infection with beta-hemolytic streptococci Group A (Strep. Pyogenes). Laboratory tests in the ED showed an elevated white blood cell (WBC) count, erythrocyte sedimentation rate (ESR), and C-reactive protein (CRP) levels (Table 1). Computer tomography (CT) imaging confirmed acute sinusitis most severe on the left side, raising suspicion of a bacterial infection (Fig. 1).

**Table 1:** Laboratory results on day of ED admission

Laboratory Results (Admission Day)	Results	Normal Range
White Blood Cell (WBC) count	**21.8**	4.2–9.4 10*3/uL
Red Blood Cell (RBC) count	4.56	3.90–4.90 10*6/uL
Hemoglobin (Hb)	13	10.8–13.3 g/dL
Platelet Count	251	150–400 10*3/uL
Neutrophil (%)	**87**	39–74%
ESR	**90**	0–20 mm/hr
CRP	**269.1**	0–5 mg/L
D-dimer	**1**,**665**	< 230 ng/mL DDU
Gram Stain	Gram (+) Cocci (Pairs)	
Blood culture	Beta-Hemolytic Streptococci Group A (Strep. pyogenes)	

**Fig. 2:**
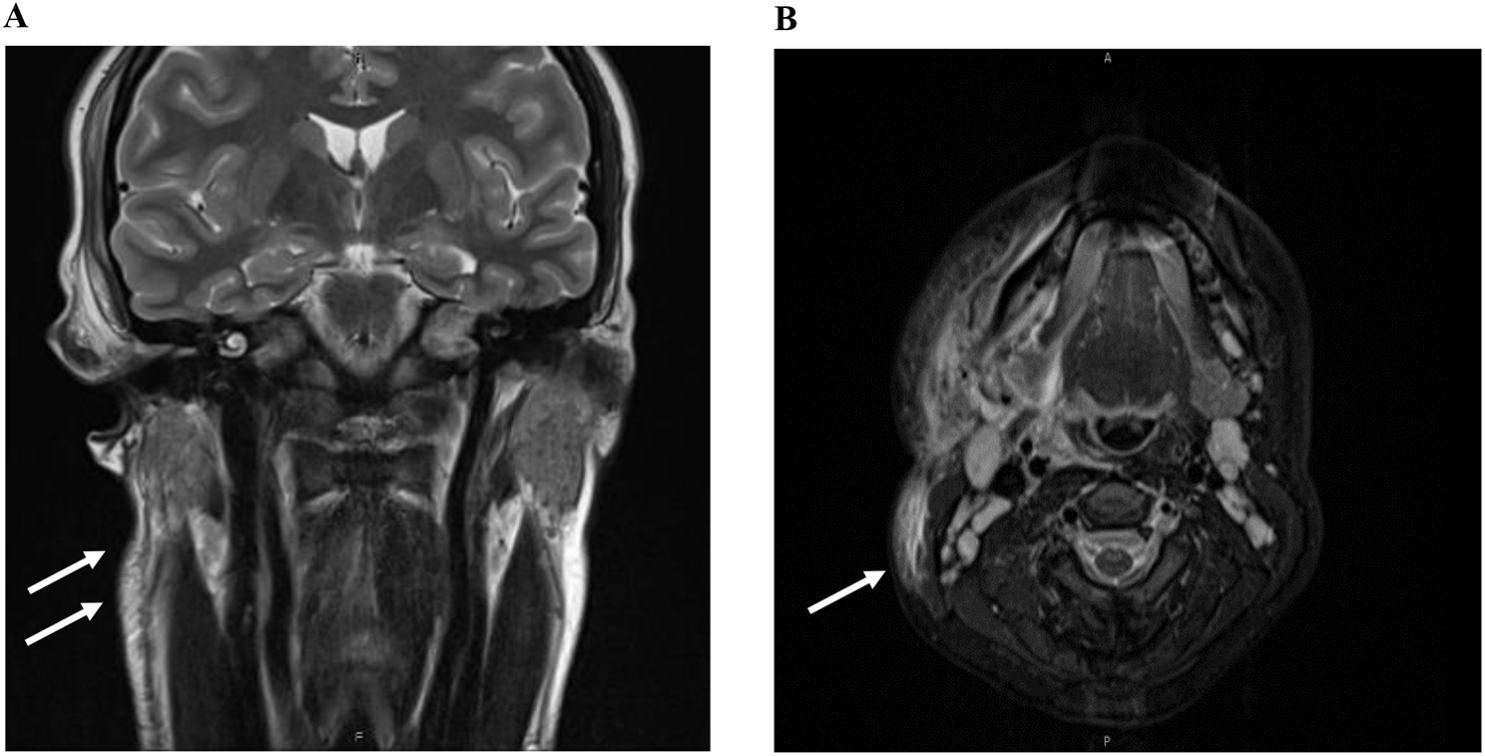
MRI sinuses without contrast showing white arrows pointing right sided myositis and parotitis in coronal (**A**) and right sided EJV thrombophlebitis in axial images (**B**)

Physical examination revealed purulence in both nasal cavities, edema of the right temporalis muscle, allodynia over the right side of the face, and oropharyngeal exudate. She did not endorse vision changes, rash, dental pain, or pain with eye movements. On the second day of hospitalization, MRI findings indicated the possibility of intracranial dural/leptomeningeal inflammation, left sinusitis, right myositis, and right parotitis (Fig. 2). Treatment included the administration of ampicillin/sulbactam, analgesics, dexamethasone, and non-steroidal anti-inflammatory drugs (NSAIDs). Additionally, blood and sinus cultures were collected for further analysis.

Blood cultures confirmed the presence of a group A Streptococcus (GAS) infection caused by Streptococcus pyogenes, which raised suspicion of Lemierre’s syndrome. Consequently, the antibiotic treatment regimen was adjusted to include ceftriaxone and clindamycin for anaerobic coverage. She remained afebrile after hospital day 2. A Doppler ultrasound revealed a non-occlusive thrombus in the right external jugular vein (EJV), while the internal jugular vein (IJV) and carotid arteries exhibited normal blood flow (Table 2). In response to the EJV thrombophlebitis, the treatment plan was further adjusted, and anticoagulation therapy with enoxaparin sodium was initiated.

During hospitalization, the patient experienced a desaturation event of 82% on room air, tachycardia, and tachypnea, prompting the use of a high-flow nasal cannula (20 L, 40%). Given the suspicion of pulmonary embolism, chest imaging was conducted on the third day of admission. Pleural effusions and atelectasis were observed on the chest X-ray, but a CT pulmonary angiogram did not reveal any evidence of a pulmonary embolism. The patient was transferred to the PICU due to a need for increased respiratory support and after stabilization, received high-dose intravenous 10 mg dexamethasone as her clinical decline was thought to be related to the worsening of the spreading inflammatory process across her face. After two days, she was transferred back to the floor with decreased facial swelling, resolution of pain, and resolution of respiratory symptoms.

Follow-up ultrasound imaging on the sixth day demonstrated an improvement in thrombophlebitis, cellulitis, and sinus disease. Of note, the patient had no mention of central line placement in the neck in her medical history and did not receive a central line during this admission. Once stable, the patient was discharged on enoxaparin therapy for 6 weeks before starting rivaroxaban daily for 4 weeks as it poses a reduced risk of bleeding in children [3]. The patient continued 4 weeks of IV antibiotics followed by 2 weeks of oral Augmentin. At 6-month follow-up, an ultrasound examination revealed no signs of acute deep or superficial vein thrombosis in the right EJV and other upper extremity vessels (Table 2). Anticoagulation treatment was discontinued 10 weeks after discharge. The patient’s juvenile idiopathic arthritis is in clinical remission, taking NSAIDS as needed. Follow-up is now on a yearly or as needed basis, and the patient no longer takes anticoagulation. Her long-term prognosis is excellent and there are no reported complications or recurrences in the medical record.

**Table 2:** Diagnostic studies and results during hospital course and at follow up

Day of Hospitalization	Diagnostic Imaging	Findings
1	CT Head and Neck	Sinusitis predominately in left frontal and paranasal sinuses
2	Bilateral US Duplex carotid arteries	No evidence of stenosis in both carotid arteries
2	Bilateral US Upper Extremities Venous	No evidence of thrombosis in bilateral internal jugular veins
2	MRI Brain and Neck with and w/o contrast	Dural and leptomeningeal enhancement likely a severe complication of sinusitis; severe sinonasal changes on the left side and right sided intracranial involvement; widespread transspatial inflammatory changes in right face, multifocal myositis and parotitis of the right side
3	MRI Sinuses w/o IV contrast	Inflammation of right periauricular soft tissue and cellulitis at right lower face; Spread of inflammation on right side of face, increased from previous MRI neck. Persistent linear T2 hypointense signal along the right external jugular vein concerning for thrombophlebitis.
3	CT Angio chest with IV contrast	No evidence of pulmonary embolism
4	Right US Duplex Upper Extremities Venous	No evidence of right internal jugular vein thrombosis; Right external Jugular Vein is dilated and non-compressible (above clavicle to mid neck)
8	MRI sinuses w/o IV contrast	Resolution of myositis; resolution of neck cellulitis; Decreased sinusitis; trace bilateral pleural effusions
10	Right US Duplex Upper Extremities Venous	Evidence of DVT in right external jugular vein
1 month followup	Right US Duplex Upper Extremities Venous	Evidence of DVT in right external jugular vein, no change from previous
6 month followup	Right US Duplex Upper Extremities Venous	No evidence of acute deep or superficial vein thrombosis in the upper extremity. Patent and compressible veins.

## Review of the literature

A PubMed search was conducted using the terms “external jugular vein” OR “external jugular thrombophlebitis,” resulting in a total of 368 articles. Non-English articles were excluded from the search, as were articles that did not specifically focus on patients with isolated external jugular vein involvement. A comprehensive literature review identified 33 other studies (*n* = 37 cases) discussing isolated EJV thrombosis or thrombophlebitis (Table 3). Among these cases, most were associated with infectious causes. Notably, a study conducted by Schwartz et al. focusing on infections involving the EJV identified 21 relevant studies (*n* = 16 cases) [4]. In contrast, our literature review diverges from their specific focus and encompasses patients with isolated EJV thrombophlebitis attributed to various etiologies. After reviewing the abstracts and references, a total of 33 articles comprising solely case reports or case series were identified (Table 3).

**Table 3:** Clinical details of 33 studies, including the present case, with isolated EJV thrombosis in the literature

Author	Reference	Patient	Age	Sex	Jugular Thrombosis	Etiology	Organism	Chief Complaint	Complications	Treatment	Length of Treatment	Resolution
Bahuth et al.	[3]	1	69	F	EJV only (extension into subclavian vein to the angle of the mandible)	-	-	swollen and tender neck	-	surgical excision	-	n/a
Colmina et al.	[1]	2	40	F	EJV only	stasis	n/a	right cervical pain, odynophagia	-	heparin	10 days	fully resolved
Cupit-Link et al.	[4]	3	18	M	EJV only	-	-	-	pulmonary septic emboli, pneumonia	anticoagulation (LMWH)	n/a	fully resolved
		4	32	M	EJV only	-	-	-	pulmonary septic emboli, neck abcess	anticoagulation (LMWH and warfarin)	n/a	fully resolved
		5	42	M	EJV only	-	-	-		conservative treatment	-	fully resolved
Ezeh et al. (current study)		6	13	F	EJV only	strep throat	Group A Streptococcus	facial swelling, cough, throat pain	sinusitis, facial cellulitis	anticoagulants (LMWH, enoxaparin) and antibiotics (ceftriaxone, flagyl, vancomycin)	6 months	fully resolved
Fishman et al.	[5]	7	37	F	EJV only	catheterization	-	pain	-	conservative treatment	-	fully resolved
Gale et al.	[6]	8	87	F	EJV only	fracture and immobilization	n/a	pain, arm pitting edema	-	anticoagulants (warfarin, enoxaparin)	3 months	fully resolved
Gonzalez et al.	[7]	9	56	M	EJV only	TMJ arthroscopy	-	neck pain	-	anticoagulants (rivaroxaban)	6 months	fully resolved
Hagiya et al.	[8]	10	74	F	EJV only	Lemierre syndrome (laryngopharyngitis)	Fusobacterium nucleatum	pharyngeal pain, cold symptoms	DIC	antibiotics (ampicillin/sulbactam, ceftriaxone/clindamycin) and anticoagulants (heparin, enoxaparin)	antibiotics (4 weeks), anticoagulants (unknown)	fully resolved
Hindi et al.	[9]	11	21	M	EJV only (bilaterally)		-	painless neck swellings, facial swelling	-	anticoagulation (heparin, warfarin)	-	fully resolved
Hulinsky et al.	[10]	12	27	F	EJV only (extension into subclavian vein to the angle of the mandible)	ovarian hyperstimulation syndrome	-	supraclavicular pain	-	anticoagulants (LMWH)	-	n/a
Hutson et al.	[11]	13	12	M	EJV only	Lemierre syndrome (secondary to tonsillitis)	-	unilateral face and neck swelling, sore throat and dysphagia	-	antibiotics (penicillin, metronidazole) and dexamethasone	7 weeks	fully resolved
Ioanno et al.	[12]	14	40	F	EJV only	aneurysm	-	tender mass in cervical region	-	surgical excision	n/a	fully resolved
Judd et al.	[13]	15	14	F	EJV only	Lemierre syndrome (secondary to tonsillitis)	n/a	neck pain, lethargy, sore throat, trismus	pulmonary septic emboli	Antibiotics (clindamycin, co-amoxyclav) and anticoagulation (LMWH, warfarin), surgical excision	antibiotics (14 days); anticoagulation (6 weeks)	fully resolved
Kim et al.	[14]	16	46	F	EJV only	aneurysm	-	n/a	-	anticoagulants (LMWH)	-	fully resolved
Lu et al.	[15]	17	19	F	EJV only	Lemierre syndrome	Fusobacterium necrophorum	tender lymphadenopathy, pleuritic chest pain, dyspnea	pulmonary septic emboli	antibiotics (piperacillin-tazobactam, metronidazole)	6 weeks	fully resolved
Morris et al.	[16]	18	18	F	EJV only	Lemierre syndrome (secondary to tonsillitis)	n/a	sore throat, neck pain, malaise, maculopapular rash	pulmonary septic emboli	antibiotics (clindamycin, ciprofloxacin) and anticoagulants (LMWH)	antibiotics (4 weeks)	fully resolved
Pucci et al.	[17]	19	59	F	EJV only	neck mass	-	neck sorness	-	surgical excision	-	-
		20	69	F	EJV only	neck mass	-		-	surgical excision	-	-
		21	24	M	EJV only	neck mass	-		-	surgical excision	-	-
Quinn et al.	[18]	22	45	F	EJV only	-	-	pain neck	-	surgical excision	-	-
Raju et al.	[19]	23	85	M	EJV only	neck trauma	n/a	neck swelling		conservative treatment	n/a	fully resolved
Ramirez et al.	[20]	24	16	M	EJV only	Lemierre syndrome	n/a	pharyngitis, weight loss, fever, should, elbow, knee pain, fatigue	-	Antibiotics (clindamycin) and anticoagulation (IV heparin, enoxaparin)	8 weeks	n/a
Reicher et al.	[21]	25	65	F	EJV only	Lemierre syndrome (secondary to tonsillitis)	n/a	sore throat, neck pain, fever, trismus	pulmonary septic emboli	antibiotics (co-amoxiclav) and anticoagulants (heparin, and oral anticoagulant)	antibiotics (4 weeks) and anticoagulation (4 weeks)	fully resolved
Safadi et al.	[22]	26	93	F	EJV only	thyroid mass	n/a	pain, redness, neck swelling	-	anticoagulants (unspecified)	n/a	fully resolved
Sanivarapu et al.	[23]	27	31	M	EJV only (extension into subclavian vein, brachiocephalic vein, superior mediastinum)	Lemierre syndrome (secondary to COVID-19)	-	neck pain, swelling	pulmonary septic emboli, parotitis	antibiotics (linezolid, piperacillin and tazobactam, clindamycin, ertapenem) and anticoagulation (apixaban)	antibiotics (4 weeks) and anticoagulation (4 weeks)	partially resolved (developed parotitis)
Schwartz et al.	[24]	28	49	M	EJV only	gingiva trauma	Klebsiella pneumoniae	fever, chills, face and neck swelling	-	antibiotics (cefotaxime, metronidazole, ciprofloxacin), anticoagulation (unspecified) and surgical excision	antibiotics (2 weeks)	fully resolved
Schwarz et al.	[25]	29	17	F	EJV only	Lemierre syndrome (secondary to tonsillitis)	n/a	chest pain, fever, mandibular tenderness, trismus, lymphadenitis, dyspnea	-	antibiotics (amoxicillin-clavulanic acid, ceftriaxone, metronidazole) and anticoagulation (enoxaparin)	n/a	fully resolved
Sengupta et al.	[26]	30	45	M	bilateral EJV	idiopathic	-	face puffiness, neck swelling	-	anticoagulation (aspirin)	n/a	fully resolved
Suzuki et al.	[27]	31	85	F	EJV only	Lemierre syndrome	Streptococcus intermedius	n/a	-	antibiotics (clindamycin, tazobactam, piperacillin) anticoagulation (heparin, edoxaban solilate hydrate)	antibiotics (37 days) and anticoagulation (37 days)	fully resolved
Suzuki et al.	[28]	32	41	F	EJV only	Lemierre syndrome	alpha-hemolytic Streptococcus	mandibular pain, toothache, trismus, fever, chills	-	antibiotics (ampicillin sulbactam)	antibiotics (4 weeks)	fully resolved
Takiguchi et al.	[29]	33	51	F	EJV only	Lemierre syndrome	group C Streptococcus	sore throat, jaw pain	shock, multiple organ failure, pulmonary septic emboli	antibiotics	n/a	fully resolved
Verma et al.	[30]	34	45	F	EJV only	aneurysm	-	progressive swelling in supraclavicular region, pain	-	surgical excision	-	n/a
Villanueva et al.	[31]	35	69	F	EJV only	idiopathic	-	painful lump in cervical region	-	anticoagulation (unspecified)	n/a	fully resolved
Warabi et al.	[32]	36	59	M	EJV only (with peritonsillar vein involvement)	Lemierre syndrome	n/a	headaches, fever, chills, temporal brain	pulmonary septic emboli, brain abscess	antibiotics (cefazolin, clindamycin) and anticoagulation (heparin)	n/a	partially resolved (developed facial nerve palsy, hearing disturbance)
Williams et al.	[33]	37	19	M	EJV only	Lemierre syndrome	Fusobacterium nucleatum	fever, sore throat, chest pain, rigors, nausea, vomiting	pulmonary septic emboli	antibiotics (levofloxacin, metronidazole) and anticoagulation (heparin)	n/a	n/a
Young et al.	[34]	38	15	F	EJV only	Lemierre syndrome	Fusobacterium necrophorum	fever, joint pain, dyspnea, throat pain, lymphadenopathy		Antibiotics (clindamycin) and anticoagulation (IV heparin, enoxaparin)	antibiotics (28 days), unknown anticoagulation	fully resolved

## Discussion and conclusions

This report presents a rare case of isolated EJV thrombophlebitis as a complication of acute pharyngitis. The diagnosis and management of our patient posed particular challenges due to concurrent sinusitis, which appeared more severe on the left side of the face based on CT imaging, while the EJV thrombophlebitis, myositis, and parotitis affected the right side of the face. Furthermore, initial concern for meningitis arose based on CT and MRI findings.

The external jugular veins are positioned laterally and superficially to the internal jugular veins. This anatomical arrangement, coupled with the common clinical utilization of the IJV as a route for accessing central circulation, likely contributes to a higher number of reported cases of IJV thrombosis compared to EJV thrombosis. Isolated thrombosis of the EJV is infrequently discussed in the literature, especially in the pediatric population [2, 5–7]. It is associated with head and neck infections [8–22], trauma [23], catheterization [24], tumor compression [25, 26], aneurysms [27, 28], or other unknown factors [5, 29, 30]. Less common factors, including obesity, orthopedic fractures, procedural complications, deep tissue massage, COVID-19 infection, and ovarian hyperstimulation syndrome, have also been implicated in EJV cases in adults [2, 23, 31–34]. Symptoms of EJV thrombosis include fever, neck pain and swelling, and sore throat [35].

The mechanism by which a head and neck infection selectively invades the EJV is not fully understood, considering its anatomical distance from the pharyngeal space compared to the IJV. Some studies have suggested that anatomical variations in the jugular venous system might contribute to this observation [4, 36, 37]. During our investigation into the underlying cause of thrombosis in our patient, the care team deliberated on the potential presence of Lemierre’s syndrome. However, the absence of typical symptoms and sequelae, such as pulmonary septic emboli, the identification of GAS as the cultured organism, and the isolated involvement of the EJV, made the diagnosis less likely at that time. Nevertheless, it is worth noting that the literature has documented instances of Lemierre’s syndrome solely affecting the EJV and involving other organisms besides Fusobacterium species (i.e. Streptococcus, Klebsiella) [19, 37]. Regardless, it is important to emphasize that this hypothetical diagnosis would not have affected the treatment course administered. Our leading hypothesis regarding the mechanism of EJV thrombophlebitis in our patient is that the barotrauma she experienced while traveling may have facilitated the spread of pharyngitis into the EJV. Existing literature supports the notion that patients with autoimmune conditions, notably juvenile rheumatic arthritis, are at a higher risk of VTE [38]. Furthermore, airplane travel has been associated with an increased risk of VTE, with a dose relationship starting at 4 h [39]. Consequently, the combination of these conditions in our patient could potentially elevate the risk of a thrombotic event. However, whether these factors also impact a more superficial vein such as the EJV remains an area of ongoing investigation.

Anticoagulation is considered the mainstay of treatment for internal jugular vein (IJV) thrombosis, while cases of IJV thrombosis resulting from infectious causes, such as Lemierre’s syndrome, require antibiotic therapy. The use of anticoagulation therapy in Lemierre’s syndrome remains a topic of debate and is typically recommended in specific situations. These include when the thrombus extends into the cerebral sinuses, in the presence of a large or bilateral clot burden, or when there is a lack of improvement despite appropriate antibiotic or surgical therapy [40]. A case report and review of the literature published in 2021 found that there is no consensus regarding the use of anticoagulation external jugular venous thrombosis [4]. However, a review found that 90% of pediatric patients who were given anticoagulation with low molecular weight heparin had thrombus improvement for resolution within a median of 3.4 months, and there were no adverse effects from anticoagulation therapy [41]. Our case was like other reported cases in the use of multimodal treatment to treat this condition involving antibiotics, anticoagulation, and fellowship trained physicians. Our case was unique in using oral anticoagulation agents, aligning with more up to date literature showing efficacy in Phase 2 trials.

In contrast, the treatment of EJV thrombosis or thrombophlebitis lacks consensus, offering a range of options that include antibiotics, anticoagulants either alone or in combination, surgical excision, or conservative management [42]. Several cases of EJV thrombosis cited in this study did use anticoagulation [4, 6, 9, 11, 13–16, 18, 20–23, 25, 27, 29–31, 33, 34, 42]. However, the use of anticoagulants remains controversial due to potential risks such as hemorrhage, thrombocytopenia, and skin necrosis. These risks must be carefully weighed against the potential of a fatal thromboembolic event.

Pulmonary embolism has been reported as a complication in approximately 10.3% of cases of internal jugular vein thrombosis [43]. Alternatively, a few documented cases of EJV thrombosis with clot propagation to the upper extremities and pulmonary vasculature exist [23, 44]. The reported number of PE complications in EJV thrombosis remains unknown, likely due to its rare occurrence. The EJV possesses a valve at its terminal end before entering the subclavian vein, preventing the regurgitation of blood from the subclavian vein to the EJV, which operates at relatively lower pressure [45]. In contrast, the internal jugular vein terminates in the brachiocephalic vein and subsequently empties directly into the superior vena cava (SVC). These anatomical considerations might explain the comparatively lower risk of pulmonary embolism in external jugular vein thrombosis when compared to internal jugular vein thrombosis. Given these distinctions, anticoagulation for EJT may not be necessary. Nevertheless, we encourage further research into this matter to gain a more comprehensive understanding of the appropriate management of EJV thrombosis.

Our study provides valuable insights into the unusual progression of an oropharyngeal infection that was appropriately treated to the severe complication of jugular embolism. EJV thrombophlebitis is a rare occurrence but our findings, supported by a comprehensive literature review, underscore the importance of heightened vigilance and clinical awareness among healthcare professionals when evaluating patients with oropharyngeal infections refractory to antibiotic treatment, and additional symptoms such as neck pain, headache, swelling, erythema, and a palpable neck mass. Prompt and accurate diagnosis is essential for effective management and the prevention of further complications. The use of anticoagulation is controversial, and the risk of embolism is far less clear with external jugular vein thrombosis [32]. Optimal treatment for EJV thrombosis has not been assessed, yet anticoagulation is likely not necessary unless the patient exhibits a severe infection or there is evidence of thrombus propagation. Further research is warranted to better understand the mechanisms underlying the selective invasion of the EJV by head and neck infections.

## Data Availability

The datasets used and/or analyzed during the current study are available from the corresponding author on reasonable request.

## Abbreviations

EJT: External jugular vein thrombophlebitis
URI: Upper respiratory infection
NSAIDs: Non-steroidal anti-inflammatory drugs
IJV: Internal jugular vein
EJV: External jugular vein
GAS: Group A streptococcus
